# What Goes on in This House Do Not Stay in This House: Family Variables Related to Adolescent-to-Parent Offenses

**DOI:** 10.3389/fpsyg.2020.581761

**Published:** 2020-12-07

**Authors:** Antonia Hernández, Ana M. Martín, Stephany Hess-Medler, Juan García-García

**Affiliations:** ^1^Fundación Canaria de Juventud Ideo, Santa Cruz de Tenerife, Spain; ^2^Departamento de Psicología Cognitiva, Social y Oganizacional, Universidad de La Laguna, San Cristóbal de La Laguna, Spain; ^3^Departamento de Psicología Clínica, Psicobiología y Metodología, Universidad de La Laguna, San Cristóbal de La Laguna, Spain; ^4^Centro de Investigación en Salud y Departamento de Psicología, Universidad de Almería, Almería, Spain

**Keywords:** adolescent-to-parent violence, exposure to violence, marital conflicts solution tactics scale, self-concept, youth offenders

## Abstract

Research on adolescent-to-parent violence (APV) associates specific psychosocial characteristics with adolescents who assault their parents, whether they are within or outside the juvenile justice system, or whether these characteristics are shared by other adolescents convicted of other crimes. The aim of this paper is to compare three groups of adolescents. Those who have been sentenced for APV are compared with adolescents who have committed other crimes, and with a group who have not been involved in the justice system. The sample used consists of 148 male participants between the ages of 14 and 21. A comparison is made regarding type of self-reported behavior, frequency of drug use, academic performance, exposure to violence, self-concept, and parents’ conflict resolution tactics. The results obtained indicate that adolescents with judicial measures, regardless of the crime committed, differ from those who have not been in trouble with the justice system in terms of them having suffered violence in the street, the frequency with which they use drugs and in their academic achievement. Likewise, adolescents convicted of APV differ from the other two groups in the frequency with which they are victims of violence at home, in that their mothers use the tactic of asking somebody else for help as a way of solving marital conflicts, and in having a more negative family self-concept. The results are discussed highlighting the importance of taking into account whether a sample is judicial, clinical, or community, and the specific APV behaviors which are measured.

## Introduction

Adolescent-to-parent violence (APV) is a type of domestic violence with very specific characteristics. It occurs in the intimacy of the home but, unlike violence from parents to children or from men to their intimate partners, it has been the target of much social reproach that has been reflected in the law since the Code of Hammurabi (Calvete and Pereira, 2019a). This reproach has given social and legal support to parents to report their children when they are their victims. However, parents have always been reluctant to do so (Williams et al., 2016). Because parents are legally and morally responsible for the children who abuse them, they experience conflicting emotions that lead them to blame themselves for what happened and remain silent because of shame (Brule and Eckstein, 2016; Williams et al., 2016). These feelings arise in the context of a parent-blaming culture in which APV is considered a failure of parenting (Holt and Retford, 2013; Holt, 2016).

Even so, in recent years, there has been an increase in the number of police reports made by parents that have brought their children into the juvenile justice and child protection systems in several countries. In Australia, for example, a 71.17% increase in APV reports to the police took place between 2009 and 2013 (Moulds et al., 2018). In Spain, references to the increase in cases of youths being prosecuted for this crime first appeared in 2004 (Calvete and Pereira, 2019a) and in 2019 Spain’s General Attorney’s Office reflected in its report the concern regarding an increase of 9.98% of these cases between 2016 and 2018 (Memoria de la Fiscalía General del Estado, 2019, pp. 891–892), as well as the lack of research that could point to possible solutions. This increase has not gone unnoticed by professionals and investigators.

Since the first explicit reference to APV was made in a scientific publication in 1957 (Sears et al., 1957), research has been directed primarily at establishing the prevalence of the phenomenon, developing measurement instruments, and analyzing the variables associated with this behavior (Simmons et al., 2018; Calvete and Pereira, 2019b). Studies related to interventions have also been published, although to a lesser extent (e.g., Ibabe et al., 2018; Curtis et al., 2019).

Reviews on the topic have attempted to structure the available evidence, using Bronfenbrenner’s (1979) nested ecological model of development, in ontogenetic, microsystemic, exosystemic, and macrosystemic factors (Simmons et al., 2018; Calvete and Pereira, 2019a). Most research has focused on ontogenetic factors, understood as individual variables, and has analyzed the impact of gender, age, patterns of antisocial behavior, and psychological factors. Psychological factors have included the use of maladaptive schemas, impulsivity, anger traits, emotional regulation, coping skills, empathy, narcissism, self-esteem, mental health, and substance abuse. Research on the microsystem has focused on interpersonal relationships, primarily on family variables, including exposure to violence, parenting styles, interpersonal conflict, and parent characteristics, such as irritability and impulsivity (Calvete and Pereira, 2019b; Gallego et al., 2019; Hoyo-Bilbao et al., 2020).

Research on exosystemic variables addresses race, socio-economic status, family structure, and school attachment (Simmons et al., 2018; Hoyo-Bilbao et al., 2020). As for the macro system, although there is little research on this topic, APV is increasingly being conceptualized as a social problem (Holt, 2016) and the influence of the social and normative context on this behavior is being considered (Williams et al., 2016; Simmons et al., 2019a,b).

The results of the research conducted so far vary depending on the APV behaviors being measured, the methodology used and the sample with which the study has been conducted (clinical, judicial, community) (Hong et al., 2012; Simmons et al., 2018; Gallego et al., 2019). In some cases, it is not clear whether the characteristics associated with adolescents who assault their parents appear regardless of whether they are within or outside the juvenile justice system, and/or whether these characteristics are shared by other adolescents serving sentences for other crimes.

### Studies With Judicial Samples

Most of the studies with judicial samples are based on an analysis of the files of youths in judicial measures, comparing those convicted of APV with those convicted of other crimes (Ibabe et al., 2009; Contreras and Cano, 2014a; Armstrong et al., 2018). From these comparisons, it has been concluded that the percentage of boys who are sentenced to prison for this offense is much higher than that of girls, and that they tend to enter the system at a later age than for other crimes. Other characteristics that are reflected in these files are drug use, mental health symptoms, behavioral problems at school, previous criminal behavior, and being part of single-parent families (Armstrong et al., 2018). However, there are studies in which youths convicted of APV are no different from those convicted of other drug offenses (Ibabe et al., 2009; Contreras and Cano, 2014a).

With regard to family structure, it should be noted that, although there are more single-parent families in the group of youths with judicial measures for APV offenses, the most frequent type of family in all cases is the traditional one, in which both parents are present (Ibabe et al., 2009; Contreras and Cano, 2014a). Single-parent families are often the result of divorce and the parent present is usually the mother (Ibabe et al., 2009; Contreras and Cano, 2014a). Contreras and Cano (2014a) warn, following Pagani et al. (2003), that the problem is not so much divorce or single parenting but the existence of a stressful family situation. This pattern is similar in the case of socioeconomic status because most families in both groups are middle or lower class, although the percentage of upper class families is higher among youths sentenced for APV than in the other group (Contreras and Cano, 2014a).

When compared with adolescents who are sentenced for other crimes, youths who have committed APV offenses are more likely to have conflictive family interactions in which violent episodes occur between parents, and between other siblings and parents (Contreras and Cano, 2014a). It has also been found that 80% of youths with judicial measures have been direct or indirect victims of domestic violence and show higher levels of aggression than those who have committed other offenses (Ibabe et al., 2009). Among adolescents incarcerated for APV offenses, there is also a higher level of physical and sexual victimization in girls than in boys, which has led to the suggestion that girls’ violence against parents is more reactive than proactive (Armstrong et al., 2018). In addition to having experienced a history of previous domestic violence, adolescents convicted of APV are often firstborn and have permissive parents (Ibabe et al., 2009; Contreras and Cano, 2014a).

When the study includes, in addition to youths convicted only of APV or only of other offenses, a third group of youths who have committed APV and other offenses are the ones who are most different from the rest (Ibabe et al., 2009). These youths are more often the firstborn, come from traditional families, have had more problems at school (adaptation, disruptive behaviors, and learning difficulties), have low self-esteem, and receive individual and family treatment. Those who have only committed APV offenses are older, come from single-parent families, have a higher social and economic status, have fewer APV offenses in their criminal record, and show less personal autonomy (Ibabe et al., 2009).

The results from the study of files described so far should be viewed with caution for several reasons. First, the information which the researcher uses has been collected for other purposes and is reflected in the files in qualitative terms or, at best, in a dichotomous manner (yes/no). Second, the professionals in charge of writing up each file may have different professional backgrounds (psychologists, educators, social workers), and the assessments they make are clinical judgments usually based on semi-structured interviews and not from assessment instruments based on evidence (e.g., self-esteem assessment). Third, at the time the assessment of the adolescent is made, the evaluators know the offense the adolescent is accused of so their expectations may have a significant effect on their assessment (Vilariño et al., 2013).

Some studies directly evaluate youths with judicial measures with questionnaires, comparing a group convicted of APV offenses with another group that has committed other offenses. In some cases, these two groups are compared with a third group of non-offenders (Contreras and Cano, 2014b, 2015, 2016a,b; Ibabe et al., 2014). In making such comparisons, it has been found that adolescents who have committed APV offenses have a higher level of school maladjustment (indiscipline, teacher rejection) and social maladjustment (aggression) than the other two groups (Ibabe et al., 2014). They share with the group of adolescents who have committed other offenses, drug use, hyperactivity, attention deficit, rule breaking, and social maladjustment. They differ, however, in that they have higher levels of personal maladjustment associated with depressive symptoms, such as affective depression, self-punishment, and low academic performance. No statistically significant differences were found in relation to self-esteem (Ibabe et al., 2014).

Youths who are prosecuted for APV also have a different family structure and dynamics than those who commit other offenses (Kennedy et al., 2010; Contreras and Cano, 2014b). They are more likely to belong to single-parent families and to have more negative parent–child relationships in which communication problems are common. They perceive their parents, especially mothers, as less loving, more critical, more rejecting, and more permissive-negligent (Contreras and Cano, 2014b).

Contreras and Cano (2015) found, as did Ibabe et al. (2014), that drug problems are common among adolescents with judicial measures, whether they have committed APV or other offenses, and that there are no statistically significant differences in self-esteem. These authors also reported similarities between both groups in impulsiveness, insensitivity to other people’s needs and less ability to perceive and retain social information. The fundamental difference between adolescents with judicial measures for APV was that they perceived their parents as more hostile and less democratic at home, and that they were less able to anticipate the consequences of their behavior and to select appropriate means to achieve their social goals.

In a later study, Contreras and Cano (2016a) found that youths with judicial measures for APV offenses were more exposed to direct and indirect violence in their home than those who had committed other offenses, whereas the latter had experienced more violence in the street than the former. Both groups reported seeing or suffering more violence in general than non-offenders. It has also been shown that adolescents who have committed APV offenses have less prosocial and more antisocial attitudes, lower emotional intelligence, and higher levels of hedonism and power as a value (Contreras and Cano, 2016b).

### The Present Study

The purpose of this study is to analyze the differences between a group of youths with judicial measures for APV offenses with a group of youths with judicial measures for other offenses, and with a third group of youths who have had no problems with the justice system, assessing directly the variables under study. In this way, the aim is to delimit which characteristics are exclusive to youths who have committed APV offenses and which are shared by youths who are serving sentences for other offenses. This general objective is specified by comparing the three groups in four sets of variables. First, they will be compared in relation to the APV behaviors they have carried out, as this type of violence is manifested through behaviors that differ in severity and frequency and which previous studies with judicial samples have not addressed. Second, the three groups will be compared in relation to drug use and academic performance, as there are discrepancies between the results of studies carried out with offenders’ files and those using direct measures.

Third, they will be compared in relation to exposure to violence, both in general terms and specifically through the marital conflict solution tactics used by parents. Previous studies are consistent that youths convicted of APV have a higher probability of belonging to families characterized by conflictive relationships and violent episodes between parents, but no studies have delved into the tactics used by these parents to deal with such conflicts in the presence of their children. The study of marital conflict solution tactics is common in the area of intimate partner relationships (Loinaz et al., 2012) and has also been used as a way to measure abuse by parents on children (Straus et al., 1998) and from children to parents (Ibabe, 2015). However, so far there are no empirical studies that analyze whether these strategies are different from those used by the rest of the parents whose children do not assault them, despite the fact that they are frequently dealt with in family intervention with parents who are victims of APV (Pérez and Pereira, 2006; Pereira, 2019).

The fourth set of variables to compare participants relates to the concept that youths who assault their parents have of themselves, since previous studies with files state that they have low self-esteem while those studies carried out with direct measures find no differences. On this occasion, we have chosen to measure self-concept, rather than self-esteem, because this is a more stable construct over time and it manifests itself in different ways in the different domains of the adolescent’s life: social, emotional, family, academic, and physical (García and Musitu, 2014). In previous studies, self-concept has been positively related to psychological adjustment (Moreno et al., 2009), and negatively to depression and anxiety (Garaigordobil and Durá, 2006), aggressive behavior (Castro-Sánchez et al., 2019), victimization (Kowalski and Limber, 2013), motives for revenge (León, 2019), and cybervictimization (Romero et al., 2019).

In addition, the relative explanatory capacity of the variables studied are assessed, when they are analyzed simultaneously, to differentiate youths who are serving sentences for APV offenses from those who are serving sentences for other offenses, and from those who have had no problems with the justice system.

## Materials and Methods

### Participants

One hundred and forty-eight young men between the ages of 14 and 21 years participated in this study (*M* = 17.21, *SD* = 1.24). There were 38 serving judicial measures for APV offenses (APV group), 52 for other offenses (Other offenses group), and the remaining 58 were students (Student group) in their first (67.2%) and second year of high school, who have not had any judicial measures against them.

In the Other offenses group, of the youths serving judicial measures, 41.2% (21) were doing so for robbery with violence and 27.5% (14) for robbery without violence. Offenses of assault and battery and forced entry were committed by three young people (5.9%); offenses of intimate-partner violence or against road safety by two young people (3.9%); and offenses of assault on authority, drug trafficking, against sexual freedom, and attempted murder by one person (2%). There was also one case (2%) that was serving the current judicial measure because of breaking a previous measure for robbery. There were 84.3% of the youths with judicial measures in the Other offenses group and 68.4% of youths in the APV group who had previous records, although the difference between the two groups was not statistically significant.

The judicial measure imposed for the majority of young people in the APV group was Living in an Educational Group (%, *n*) (60.5%, 23), followed by Open or Semi Open Imprisonment (21.1%, 8), Probation (13.2%, 5), and Weekend Imprisonment (2.6%, 1). In the case of youths in the Other offenses group, the measures were Probation (32.7%, 17), Living in an Educational Group (30.8%, 16), Open or Semi Open Imprisonment (28.8%, 15), and Attendance at a Day Center (1.9%, 1).

The number of young people diagnosed with mental illness in the sample was 6.2% (9), and the differences between the three groups in this aspect were not statistically significant. The percentage of youths who admitted to drug use was 83.8% (124) and, in this case, the differences between the groups were statistically significant [χ^2^(2) = 23.46, *p* = 0.001, Cramer’s *V* = 0.40]. The percentage of adolescents in the Student group who admitted drug use was lower (65.5%) than that of youths with judicial measures in the group of APV (94.7%) and of Other Offenses (96.2%), which did not differ significantly from each other.

The difference between the groups in Frequency of drug use was statistically significant [*F*(2, 78.79) = 58.32, *p* = 0.001, η^2^ = 0.46]. Students were those who recognized less frequent use (*M* = 2.34, *DT* = 2.28) vs. the APV group (*M* = 7.03, *DT* = 2.96) and the Other offenses group (*M* = 6.88, *DT* = 3.12), which had no statistically significant differences between them. Differences between the three groups in Academic performance were also statistically significant [*F*(2, 72.95) = 22.04, *p* = 0.001, η^2^ = 0.24] and, as expected, students were perceived to have better performance (*M* = 6.93, *DT* = 1.28) vs. the APV (*M* = 4.76, *DT* = 2.67) and Other offenses group (*M* = 4.40, *DT* = 2.87). The latter two showed no statistically significant differences.

The traditional family is the most frequent in the Student group (84.5%) and in the Other offenses group (36%), while in the APV group, it is a family structure in which the mother is alone or with a new partner (56.8%). Table 1 reflects the distribution of participants in relation to family structure.

**TABLE 1:** Participants’ distribution according to their family structure.

	Family structure				
	
	Mother	Father	Both	Others	Total
Other offenses	13	6	18	13	50
	26.0%	12.0%	36.0%	26.0%	100.0%
APV	21	2	12	2	37
	56.8%	5.4%	32.4%	5.4%	100.0%
Students	6	2	49	1	58
	10.3%	3.4%	84.5%	1.7%	100.0%
Total	40	10	79	16	145
	27.6%	6.9%	54.5%	11.0%	100.0%

### Instruments

To collect information on the variables under study, a questionnaire was prepared that included the following scales and questions.

The nine self-reported APV behaviors were measured, according to Hernández (2016), by means of the following question: “During the time living with your parents or tutors, how often do you perform or have you performed some of the following behaviors?” The participants had to answer in relation to nine items, chosen from Cottrell’s (2001) definition, which refer to behaviors aimed at controlling and/or causing physical, psychological, emotional, or economic harm to parents. These behaviors were as follows: Insulting; Running away from home; Spitting; making Obscene gestures; Stealing; Destroying their things; getting parents into Debt; Intimidating, blackmailing, or threatening them; Hitting, punching, throwing objects at them, and pushing them. Participants were asked to respond on an 11-point Likert-type scale, from 0 (Never) to 10 (Most often). Although this time the score of each item was used separately, Hernández (2016) has provided evidence of validity and reliability for the overall scale.

The Orue and Calvete Observed Violence Scale (2010) was used to measure previous violence exposure. It consists of 21 items, of which 9 relate to direct exposure as a victim and 12 to indirect exposure as a witness. In each case, the items refer to three types of violence (physical, verbal, and threats) in four contexts (school, street, home, and TV). Participants were asked to answer each item on an 11-point Likert scale from 0 (Never) to 10 (Every day). This response scale was preferred to the original one from 1 to 5 because it is more akin to the one commonly used in the Spanish educational system. Several investigations have provided evidence of validity and reliability for this scale (see Orue and Calvete, 2010). In this study, the internal consistency, measured with Cronbach’s alpha, for the different subscales was Seeing violence in the classroom 0.79, Seeing violence in the street 0.85, Seeing violence at home 0.81, Seeing violence on TV, 0.79; Suffering violence in the classroom, 0.75; Suffering violence in the street, 0.82; and Suffering violence at home, 0.72.

The Autoconcepto Forma-5 [Self-concept Form-5] (AF5) scale by García and Musitu (2014) is composed of 30 items and was used to measure five dimensions of self-concept: Social self-concept, Emotional self-concept, Family self-concept, Academic self-concept, and Physical self-concept. Participants were asked to answer each item on an 11-point Likert-type scale from 0 (Total Disagreement) to 10 (Total Agreement). This response scale was preferred to the original one from 1 to 99 because it is more akin to the Spanish educational system. Several investigations have provided evidence of validity and reliability for this scale (see García and Musitu, 2014). In this study, the internal consistency, measured by Cronbach’s alpha, was 0.89 for Academic self-concept, 0.70 for Social self-concept, 0.71 for Emotional self-concept, 0.81 for Family self-concept, and 0.79 for Physical self-concept, 0.73.

To measure the parents’ marital conflict resolution strategies, Straus’s (1979) Conflicts Tactics Scale, adapted to Spanish by Muñoz-Rivas et al. (2007), was used. Participants were asked the question: “When conflicts occur between your parents, how often do you witness the following reactions?” They were given 18 behaviors that they had to score in relation to their father and their mother, using an 11-point Likert scale from 0 (Never) to 10 (Always). These behaviors were the following: Talking quietly; Searching for information to support their point of view; Asking someone else for help; Insulting or cursing; Refusing to talk about a subject; Leaving the room upset; Crying; Saying something to annoy; Threatening to hit; Physically holding; Throwing an object; Hitting or hurting with an object; Pushing or grabbing; Slapping; Kicking, hitting, biting; Drowning; Thumping; and Threatening with a knife or other weapon.

To measure academic performance, participants were asked directly “Do you perform well academically?” providing them an 11-point Likert-type scale from 0 (Never) to 10 (Very often) to answer. In relation to drug use, participants were asked to three questions: “Do you use or have used drugs or alcohol?” “What substance?” “How often?” They were requested to answer the first question using a Yes/No scale, and the third by an 11-point Likert-type scale, from 0 (Never) to 10 (Very often). The second question was open to allow to report any possible substance.

The questionnaire also included queries about age, family structure, mental health diagnosis and, in the case of adolescents in the juvenile justice system, their legal situation, including the offense, the type of legal measures, and previous records. This information on the legal situation was checked with the persons in charge of supervising the implementation of the adolescents’ legal measures.

### Procedure

In the case of young offenders, after obtaining authorization from the government authority, the project was submitted to the heads of the entities responsible for implementing the legal measures. The technical staff of those entities were asked to obtain the informed consent of the young people and their legal guardians, and to ensure that the data collection interfered as little as possible with the functioning of the center and with the youths’ daily activities.

In the interviews with participants, they were informed of the objectives of the project, and the anonymity and confidentiality of the information they provided was reiterated. Each participant answered the questionnaire individually or in small groups at the place where they were serving the judicial measure, or at the facility of the collaborating entity when they were on probation. In cases where reading comprehension was not good, the questionnaire was administered as a structured interview. Once the questionnaire was completed, it was checked and confirmed through the judicial authority whether the young people assigned to each group had or not had measures imposed for APV.

In the case of the students, after obtaining permission from the directors of the educational centers, it was explained to the participants that a study was being carried out from the university to find out “the habits and behaviors of adolescents today, both inside and outside the home.” They were assured that their participation was anonymous and voluntary. All students agreed to participate and signed an informed consent form. Because they were all over 14 years old and outside the juvenile justice system, informed parental consent was not legally required. However, parental permission was obtained anyway in accordance with the World Medical Association’s Declaration of Helsinki. The questionnaire was answered in the classroom, during regular class hours.

### Design and Data Analysis

To carry out the research, a non-experimental design involving cross-sectional comparison between the independent groups on a series of variables was followed (Ato et al., 2013). Data analysis was conducted using the IBM SPSS 26.0 statistical package for Windows (IBM Corporation, 2019) and Real Statistics Resource Pack software 7.2 (2013-2020). First, for sample description purpose, tests of χ^2^ were carried out to check the relationship between the group of participants and the categorical variables Drug use and Diagnosis of mental illness. We also analyzed whether there were statistically significant differences between the groups in Frequency of drug use and in Academic performance through ANOVA. Second, the internal consistency of the scales was calculated using Cronbach’s alpha, the scale items were averaged to create the corresponding variables, and the descriptive analysis of all variables was performed. Cronbach’s alphas were described in the instrument section and descriptive statistics in the subsequent analyses in the result section. Third, the groups under study were compared in relation to the performance of APV behaviors by means of a MANOVA, with the Group variable as an independent variable and the nine APV behaviors as dependent variables. Fourth, four MANOVA were carried out to analyze in which variables of the four sets of exposure to violence, self-concept, and mother’s and father’s marital conflict resolution tactics the three groups of participants were significantly different, and what the effect size was in each case. For each of these MANOVA, it was previously verified that the correlations between the corresponding dependent variables were statistically significant and always between 0.2 and 0.8. Finally, a discriminant analysis was carried out to differentiate between the three groups of participants using the variables that had been statistically significant in the previous four MANOVA.

As statistical assumptions underlying the lineal model were not fully met, parameters were estimated using the resampling method bootstrapping simple and permutational under the simulation of 1,000 samples. Bootstrap bias-corrected accelerated method was used as a corrective method. The estimation of the MANOVA was carried out by means of the type III and type IV sum of squares, depending on the case, and with the estimation of Pillai’s Trace.

For univariate inter-subject tests, robust tests of equality of means were calculated using Welch’s F and for pair comparison Dunnett’s C when variances were heterogeneous. The effect size was obtained by using Partial Eta Squared for multivariate analysis, Eta Squared for univariate analysis, and Cramer’s V for χ^2^ tests. Discriminant analyses had as starting point the group different sizes and validation classification with quadratic discriminant analysis.

## Results

As described in the section on data analysis, a MANOVA was carried out using the variable Group with three levels as an inter-subject factor: young people with judicial measures for APV, young people with judicial measures for Other offenses, and Students. The dependent variables were the APV behaviors: Insulting, Running away, Obscene gestures, Spitting, Stealing, Destroying things, getting parents into Debt, Intimidating, and Hitting. The results showed multivariate statistically significant differences in APV in function of the variable Group [*Pillai’s Trace* = 0.83, *F*(18, 248) = 9.88; *p* exact < 0.001, η*_*p*_*^2^ = 0.42]. As reflected in Table 2, evidence of inter-subject effects indicated that statistically significant differences existed between the three groups in all APV behaviors.

**TABLE 2:** Robust tests of equality of means (Welch) for the nine APV behaviors.

Variables	*F*	*df*	η^2^
Insulting	28^*abc*^	2, 66.93	0.29
Running away	44.74^*bc*^	2, 53.23	0.38
Spitting	6.09^*ab*^	2, 60.98	0.02
Obscene gestures	6.02^*b*^	2, 55.27	0.06
Stealing	15.21^*abc*^	2, 50.50	0.14
Destroying	21.29^*abc*^	2, 52.75	0.14
Debts	18.05^*bc*^	2, 49.66	0.20
Intimidating	18.79^*abc*^	2, 50.15	0.17
Hitting	8.33^*abc*^	2, 49	0.08

**All F are statistically significant for p < 0.001. ^*a*^Statistical significant difference (p < 0.05) between APV and Other offenses. ^*b*^Statistical significant difference (p < 0.05) between APV and Students. ^*c*^Statistical significant differences (p < 0.05) between Other offenses and Students.**

**FIGURE 1:**
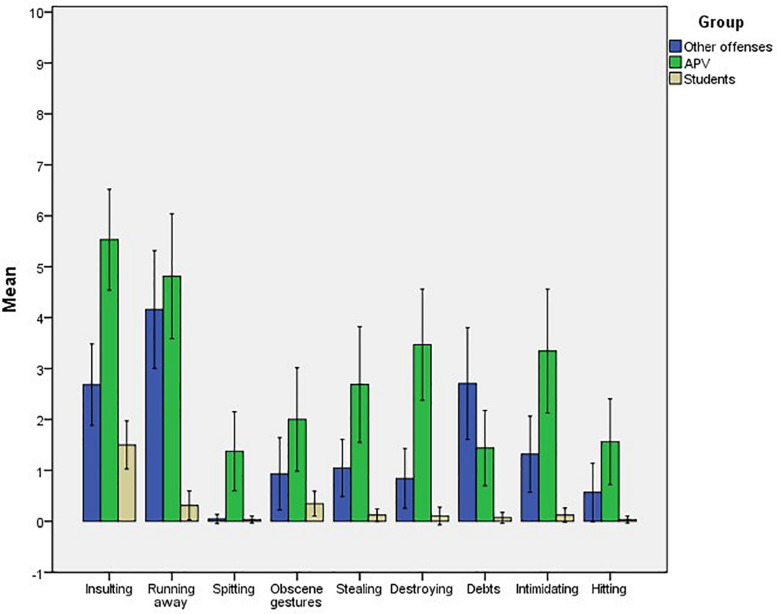
Means and confidence intervals in the nine behaviors of APV for participants in the groups of APV, Other offenses, and Students.

The effect size was small for Spitting, intermediate for making Obscene gestures and Hitting, and large for all other behaviors. A posteriori comparison tests (Dunnett’s C) established that there were statistically significant differences between the three groups in Insulting, Stealing, Destroying things, Intimidating, and Hitting. Student group reported Running away and getting parents into Debts less than the APV group and the Other offenses group, which did not differ from each other. Youths with APV measures reported more Spitting than the other two groups, which did not differ from each other. Last, Student group also reported less frequent Obscene gestures than APV; the difference between these two groups and the Other offenses group was not statistical significant. The averages of the three groups in the nine behaviors are shown in Figure 1.

Four MANOVA were then carried out to analyze which variables had statistically significant differences between the three groups of participants and to estimate the corresponding effect sizes. The dependent variables in each of them were, separately, the variables of exposure to violence, self-concept, and mother and father’s marital conflict resolution tactics. Statistically significant multivariate effects in function of the variable Group were found for exposure to violence [*Pillai’s Trace* = 0.6, *F*(12, 278) = 9.85; *p* exact < 0.001, η*_*p*_*^2^ = 0.30], self-concept [*Pillai’s Trace* = 0.26, *F*(10, 282) = 4.20; *p* exact < 0.001, η*_*p*_*^2^ = 0.13], mother’s marital conflict resolution tactics [*Pillai’s Trace* = 0.50, *F*(36, 210) = 1.95; *p* exact < 0.001, η*_*p*_*^2^ = 0.25], and father’s marital conflict resolution tactics [*Pillai’s Trace* = 0.59, *F*(36, 192) = 2.21; *p* exact < 0.001, η*_*p*_*^2^ = 0.29]. The descriptive statistics of these variables for the three groups, as well as the univariate inter-subject effects, are presented in Table 3.

**TABLE 3:** Descriptive statistics for the three groups in exposure to violence, self-concept, and mother’s and father’s marital conflict resolution tactics.

	APV		Other offenses		Students		Inter-subject tests			
				
Exposure to violence	Mean	SD	Mean	SD	Mean	SD	*F*	*df*	*p*	η^2^
Seeing violence in the classroom	5.98	2.61	5.08	2.90	3.99	1.79	9.18^*abc*^	2, 78.88	0.000	0.10
Seeing violence in the street	7.24	2.64	7.12	2.23	5.16	2.34	12.49^*bc*^	2, 85.36	0.000	0.15
Seeing violence at home	2.92	2.89	1.37	1.68	0.60	1.11	13.01^*abc*^	2, 71.69	0.000	0.10
Seeing violence on TV	7.00	2.88	6.75	2.71	6.71	2.24	0.14	2, 83.07	0.869	0.001
Suffering violence in the classroom	2.44	2.26	2.64	2.68	1.62	1.63	3.70^*c*^	2, 79.67	0.029	0.05
Suffering violence in the street	4.21	2.34	4.68	2.48	1.41	1.63	41.84^*bc*^	2, 79.72	0.000	0.10
Suffering violence at home	2.61	2.64	1.15	1.75	0.65	1.09	9.69^*ab*^	2, 71.70	0.000	0.06
**Self-concept**										
Academic self-concept	5.93	2.22	5.57	2.56	6.38	1.79	1.77	2, 81.32	0.177	0.00
Social self-concept	7.65	1.76	7.52	1.44	7.11	1.88	1.61	2, 87.42	0.205	0.00
Emotional self-concept	6.47	1.54	6.22	1.91	6.25	1.73	0.13	2, 88.96	0.88	0.00
Family self-concept	6.50	2.32	7.82	1.93	8.53	1.42	23.19^*abc*^	2, 76.79	0.000	0.10
Physical self-concept	6.64	1.42	6.89	1.91	6.73	1.74	0.20	2, 91.50	0.82	0.00
**Father’s strategies**										
Talking quietly	4.70	3.66	5.12	3.19	6.20	3.13	2.18	2, 59.79	0.122	0.02
Searching for information	4.00	3.37	3.85	3.70	4.61	3.44	0.56	2, 62.04	0.573	0.00
Asking for help	3.26	3.65	1.76	2.62	1.70	2.95	1.98	2, 59.72	0.147	0.01
Insulting/cursing	3.74	3.28	2.32	3.12	1.11	2.41	7.33^*b*^	2, 55.75	0.001	0.09
Refusing to talk	3.74	3.63	3.68	3.59	2.07	2.83	3.63	2, 56.75	0.033	0.06
Leaving the room upset	3.19	3.56	4.47	3.66	3.13	3.38	1.61	2, 60.84	0.209	0.00
Crying	2.19	2.96	2.41	3.09	0.72	1.74	6.04^*c*^	2, 50.43	0.004	0.10
Saying something to annoy	4.22	3.49	2.65	3.12	2.22	3.21	3.12^*c*^	2, 61.07	0.051	0.00
Threatening to hit	2.11	3.30	1.29	2.39	0.33	1.48	5.05^*b*^	2, 48.90	0.010	0.07
Physically holding	1.81	3.19	1.59	2.56	0.06	0.30	9.91^*bc*^	2, 39.27	0.000	0.14
Throwing an object	1.48	3.09	0.71	2.29	0.09	0.45	3.77	2, 40.02	0.032	0.04
Hitting	1.81	3.40	1.44	3.00	0.37	1.15	3.95	2, 43.98	0.026	0.06
Pushing or grabbing	2.00	3.26	1.00	2.42	0.06	0.23	7.21^*b*^	2, 39.08	0.002	0.09
Slapping	1.48	3.22	0.76	2.19	0.04	0.27	4.45	2, 39.26	0.018	0.06
Kicking	1.52	3.38	0.41	1.83	0.00	0.00	–	–	–	–
Drowning	0.74	2.18	0.29	1.71	0.00	0.00	–	–	–	–
Thumping	1.48	3.25	0.68	2.24	0.00	0.00	–	–	–	–
Threatening with a knife/weapon	1.30	2.83	0.26	1.54	0.02	0.14	3.13	2, 39	0.055	0.03
**Mother’s strategies**										
Talking quietly	4.14	3.80	5.29	3.25	5.65	2.92	1.75	2, 63.18	0.183	0.01
Searching for information	4.79	3.58	4.39	3.87	4.95	3.36	0.26	2, 66.10	0.775	0.002
Asking for help	4.52	3.71	2.32	3.07	1.75	2.63	6.33^*ab*^	2, 61.68	0.003	0.04
Insulting/cursing	3.83	3.58	1.87	2.44	1.46	2.71	4.91^*ab*^	2, 64.15	0.010	0.03
Refusing to talk	3.48	3.39	3.63	3.04	2.53	3.16	1.69	2, 67.06	0.193	0.001
Leaving the room upset	4.00	3.59	3.08	2.97	3.65	3.51	0.71	2, 68.15	0.496	0.001
Crying	5.38	3.45	3.68	3.55	2.21	2.85	9.49^*ab*^	2, 63.36	0.000	0.10
Saying something to annoy	4.03	3.20	2.79	2.92	2.54	3.11	2.19	2, 67.78	0.120	0.01
Threatening to hit	1.62	2.68	0.97	2.06	0.47	1.68	2.50	2, 59.68	0.091	0.03
Physically holding	1.59	2.60	0.92	1.92	0.35	1.48	3.37^*b*^	2, 58.08	0.041	0.03
Throwing an object	1.83	3.17	0.92	2.12	0.46	1.65	2.63	2, 57.25	0.081	0.03
Hitting	1.24	2.13	0.61	1.33	0.51	1.53	1.36	2, 63.50	0.263	0.008
Pushing or grabbing	1.17	2.02	0.66	1.46	0.32	1.47	2.19	2, 63.11	0.121	0.02
Slapping	1.21	2.08	0.66	1.49	0.21	1.21	3.38^*b*^	2, 58.72	0.041	0.03
Kicking	0.76	1.94	0.26	0.86	0.18	1.20	1.09	2, 62.05	0.343	0.00
Drowning	0.14	0.74	0.08	0.36	0.00	0.00	–	–	–	–
Thumping	0.14	0.74	0.16	0.59	0.14	1.06	0.01	2, 72.23	0.991	0.00
Threatening with a knife/weapon	0.48	1.27	0.05	0.16	0.04	0.26	1.83	2, 57.80	0.170	0.03

**APV, adolescent-to-parent violence. ^*a*^Significant difference (p < 0.05) between APV and Other offenses. ^*b*^Significant difference (p < 0.05) between APV and Students. ^*c*^Significant differences (p < 0.05) between Other offenses and Students.**

The variables that were statistically significant in these analyses, and have an effect size of η^2^ > 0.39 (intermediate effect), were introduced into a further discriminant analysis in which the classifying variable was the Group to which the participants belonged and the discriminant variables: Seeing violence in the street, Seeing violence at home, Seeing violence in the classroom, Suffering violence in the street, Suffering violence at home, Suffering violence in the classroom, Family self-concept, mother’s use of the tactics of Asking somebody else for Help and Crying, as well as father’s use of the tactics Insulting/Cursing, Refusing to talk, Crying, Threatening to hit, Physically holding, Throwing an object, Hitting, Pushing or grabbing, and Slapping. The variables Frequency of drug use and Academic achievement were also included in the discriminant analysis because the differences between the groups were statistically significant, as described in the Participant section. A step-by-step method was used with Wilk criteria and group size was taken into account to carry out the analysis.

Two statistically significant discriminant functions were obtained, which allowed 79% of the cases to be correctly classified. The rotated structure matrix indicated that only 6 of the 20 variables included in the analysis were used in the solution. Consequently, a second discriminant analysis was carried out including only these five variables: Frequency of drug use, Academic achievement, Suffering violence in the street, Suffering violence at home, and mother’s use of the tactics of Asking somebody else for Help. The percentage of cases correctly classified was the same, suggesting that this solution was more parsimonious. The cases correctly classified were validated with quadratic discriminant analysis. As reflected in Table 4, when the discriminant functions misclassify the cases in the APV group, they assign them to the Other offenses except for one participant. The misclassifications of the Other offenses cases are distributed equally in both groups of APV and Students. Finally, misclassifications of Students are mainly in favor of the Other offense group except for one participant.

**TABLE 4:** Classification results for the participants in the groups of APV, Other offenses, and Students, using the two functions of the discriminant analysis.

			**Predicted group membership**			**Total**
	
			**Other offenses**	**APV**	**Students**	
Original group membership	*n*	Other offenses	24	7	6	37
		APV	8	21	1	30
		Students	3	1	53	57
	%	Other offenses	64.9	19.9	16.2	100
		APV	26.7	70	3.3	100
		Students	5.3	1.8	93	100

**79% of original cases correctly classified.**

**FIGURE 2:**
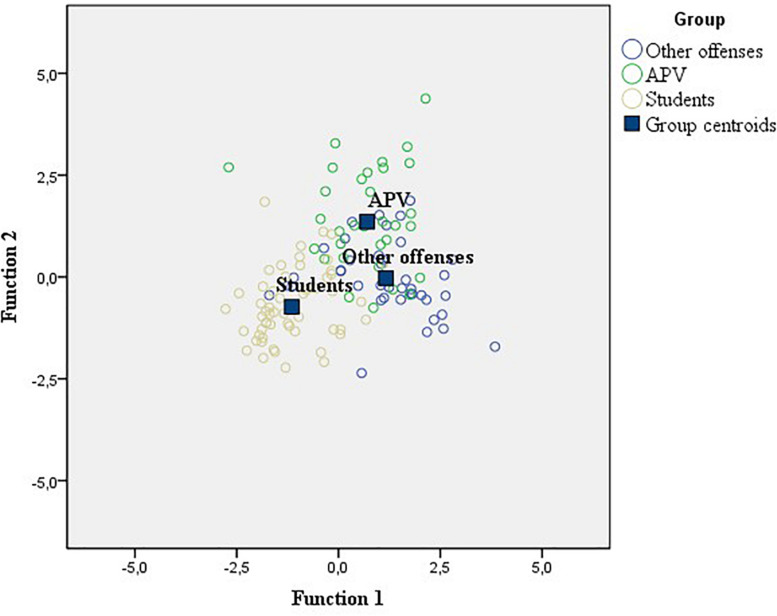
Group centroids and distribution of the participants of the groups of APV, Other offenses, and Students, according to the two discriminant functions.

Figure 2 shows that the first discriminant function [λ = 0.27, χ^2^(12) = 153.15; *p* < 0.001] places the centroids of the groups so that the Other offenses group is at one end (1.29), the Students group at the other (-1.24), and the APV group between the two (0.76), although closer to the other group of young people with judicial measures than to the Students group. The second function [λ = 0.73, χ^2^(5) = 36.82; *p* < 0.001] places the centroids of the groups so that the APV group is at one end (1.38), the Students group at the other end (-0.61), and the Other offenses group between the two (-0.18), closer to the Students than to the other group of young people with judicial measures.

The rotated structure matrix indicates that the first discriminant function is defined positively by the variables Suffering violence in the street (0.66) and Frequency of drug use (0.53), and negatively by Academic achievement (-0.39). The second function is defined positively by Suffering violence at home (0.62), mother’s use of Asking for help a tactic to solve marital conflicts (0.43) and, negatively, by the Family self-concept (-0.48). In this way, young people with judicial measures, regardless of the offense committed, differ from the Student group in the frequency with which they are victims of violence in the street, with which they acknowledge using drugs and in having a lower academic achievement. Likewise, young people in the APV group differ from the other two groups in the frequency with which they suffer violence at home, in that their mothers use the tactic of asking somebody else for help as a way of resolving marital conflicts, and in having a more negative family self-concept.

## Discussion

The aim of this study was to compare a group of young people who were sentenced for APV with a group of young people who had committed other offenses, and with a third group who had not been in trouble with the justice system. A comparison was made in relation to the type of self-reported behavior, frequency of drug use, academic performance, exposure to violence, self-concept, and parents’ marital conflict resolution tactics. The results obtained indicate, first of all, that, as expected, young people in the APV group are the ones who most often insult, destroy things, steal from, intimidate, and hit parents, although young people who have committed other offenses also do so more often than students. The two groups with legal measures do not differ from each other in running away and in getting parents into debts, and do so more often than students.

The behaviors in which the young people in the APV group differ from the others are spitting, the least frequent even in the APV group. It is also worth noting the low frequency with which students perform all the behaviors, except the one of insulting. These differences highlight the importance of taking into account the behaviors through which APV is measured when comparing judicial, clinical, and community samples, or when the study focuses exclusively on the latter. If the frequency of behaviors that differ in severity is averaged, the final score may be misleading in some cases. The Cortina and Martin study (2020) shows that behaviors, such as spitting, intimidating, and hitting never occur alone in a community sample and that insulting, which does occur, is not a valid indicator of APV, as it is now relatively common for most teenagers to shout at and insult their parents at some point.

As with violence in intimate-partner relationships, APV follows an escalation of frequency and severity into abuse and the turning point comes when parents decide to seek clinical or legal help (Holt, 2016). One of the immediate consequences of not considering APV behaviors like insulting or shouting is that the prevalence of APV in the general population is reduced (Condry and Miles, 2014; Cortina and Martín, 2020). Recently, Simmons et al. (2019b) have developed an instrument for detecting parent–child abuse by considering when the severity or frequency of violent behavior exceeds what is socially considered “normal.” The use of instruments, such as these in future research could help to clarify when a case of APV has happened, or simply when the behavior occurring is just a lack of respect, which is undesirable of course, but does not constitute abuse (Kennedy et al., 2010; Hollenstein and Lougheed, 2013; Simmons et al., 2019b).

Second, the results of this study replicate those obtained by Contreras and Cano (2016a) regarding the differences between the three groups in terms of exposure to violence and drug use. Although these authors do not refer to academic performance, the results obtained with respect to this variable are along the same lines as those relating to drug use. As with APV behaviors, exposure to violence is not the same when it occurs at home, at school, on the street or on TV, nor is it the same to be a witness or a victim. Differences were found in all forms of exposure to violence, except watching violence on TV, as all three groups scored equally high in this regard.

However, what differentiates the APV group from the other two groups is seeing and suffering violence at home, as pointed out by the bi-directionality of violence hypothesis (Brezina, 1999; Gallego et al., 2019). Both variables are highly correlated, so when analyzed together, only suffering violence at home defines the discriminant function. Moreover, the single-parent family is the most frequent in the APV group, as opposed to the traditional one in the students and other offenses groups. However, as argued by Contreras and Cano (2014a), single-parent families are usually the result of divorce and the parent present is usually the mother, so the problem is not so much the absence of the father but the existence of a stressful family situation that may well precede or parallel the marital separation. It is worth noting at this point that in the majority of the most serious cases of intimate partner violence, the aggressor is the ex-partner of the victim (Fleury et al., 2000).

Going back to the types of exposure to violence, young people with judicial measures share, in contrast to students, seeing and suffering violence in the street. As in the case of exposure to violence at home, seeing and suffering violence in the street are highly correlated so that, when analyzed together, only one of the two variables defines the discriminating function. However, these data should be viewed with caution as they come from a cross-sectional, not a longitudinal, study and therefore no causality should be inferred from them. It is possible that exposure to violence in the street as well as increased frequency of drug use are consequences of serving judicial measures and not causes of them. Alternatively, they may simply be spurious relationships that respond to the impact of some other variable that has not yet been taken into account. This caution extends to the interpretation of the higher academic performance of the student group in relation to young people with judicial measures. It is important for future research to explore this point further, on the basis that they are characteristic not so much of young people who carried out APV, but rather of young people who are in the juvenile justice system.

Third, our results regarding self-concept show that it is a more appropriate construct to be studied in relation to APV than self-esteem, given its stability over time and the possibility to differentiate between several domains of an adolescent’s life, along the lines of García and Musitu (2014). There are differences between the APV group and the other groups, but as might be expected, these differences focus only on the family facet of self-concept. It is interesting to note at this point that the facets of self-concept that were most related to cybervictimization (Romero et al., 2019) and revenge motivation (León, 2019) were family and academic. As in the case of exposure to violence in the street and frequency of drug use, the design of this study only allows us to know that there is a relationship between both variables, but not if low self-concept is a cause, as suggested by the studies focused on self-esteem, an effect, or simply a correlate of APV which underlies an adverse family context. In any case, these data should be taken into account when carrying out future research and setting the objectives of family interventions in cases of APV (Carrasco et al., 2018). It is logical to think that family self-concept is the dimension of self-concept most related to intra-family violence, not only because both refer to the same life domain but because family relationships have an important role in the origin, maintenance, and desistance of offending behavior (Martín et al., 2019).

The results also indicate that the mothers of young people in the APV group are the ones who ask somebody else for help the most, when compared with the other two groups; they are also the ones who shout and insult the most. It is reasonable to think that the strategy of asking somebody else for help is related to higher levels of intimate partner violence, with advanced stages of the violence escalation, as young people in the APV group are the ones who report being exposed to and suffering higher levels of domestic violence. Asking somebody else for help is generally considered an inadequate marital conflict resolution tactic (Straus, 1979), but may be the only way out when there has been an escalation of gender-related violence. Previous use of this strategy with a violent intimate partner may lead the mother to use it later with a son who abuses her, as a result of which he ends up in the juvenile justice system. It is likely that in community settings, mothers who are victims of APV will use other strategies, as the cases that reach the juvenile justice system are the most serious, and seeking outside help may be the result of previous failures using other strategies. This argument is supported by the fact that young people with APV offenses enter the juvenile justice system at an older age (Armstrong et al., 2018).

Although the strategy that allows discrimination between the three groups is that the mothers ask somebody else for help, it is also relevant for family interventions to analyze in more detail the type of marital conflict resolution strategies used by both parents. In this sense, the adolescents in the APV group differ from the student group, but not from the other offenses group, in that their mothers cry and slap more. In the case of parents, the differences are with students in insulting, threatening to hit, and pushing and with both groups in physically holding. Moreover, different from the students group are the young people in the other offenses group regarding saying something to annoy and crying. Curiously, it is parents of the other offenses group who seem to cry the most, above not only those in the student but in the APV group. It is interesting to note that there are no differences between the three groups in the positive strategies.

At this point, it should be noted that this work has several limitations that recommend caution in drawing conclusions. The most important is the small sample, which is due to the number of members of the APV group that was available in the territory at the time the study was conducted. In addition, although women were initially included, their relatively smaller numbers and different distribution in each group made gender comparisons unfeasible, and girls were excluded from the analyses. Unlike community samples, judicial samples are small, and the percentage of girls does not exceed 8%. To increase the number of participants, if data collection is not to be extended over time, it is necessary to have access to other territorial jurisdictions which, as in the case of this study, may be on different islands or at a distance of more than 2,000 km. To solve this difficulty, future research should promote collaboration between researchers so that samples from different territories can be integrated. Lastly, measures of social desirability should also be included, given the social reproach to which APV is subjected (Calvete and Pereira, 2019a). Moreover, the forensic context of participants may influence them to hide the negative characteristics they possess and/or simulate positive characteristics that they lack (Arce et al., 2015; Fariña et al., 2017).

## Data Availability Statement

The raw data supporting the conclusions of this article will be made available by the authors, without undue reservation.

## Ethics Statement

Ethical review and approval was not required for the study on human participants in accordance with the local legislation and institutional requirements. Written informed consent to participate in this study was provided by the participants/participants’ legal guardian/participants’ next of kin.

## Author Contributions

All authors contributed in all the phases of the research and the elaboration of the article.

## Conflict of Interest

The authors declare that the research was conducted in the absence of any commercial or financial relationships that could be construed as a potential conflict of interest.

## References

[B1] Arce R. Farina F. Vilarino M. (2015). Daño psicológico en casos de víctimas de violencia de género: Estudio comparativo de las evaluaciones forenses [Psychological damage in cases of victims of gender-based violence: a comparative study of forensic evaluations]. *Revist. Iberoameric. Psicol. Salud* 6 72–80. 10.1016/j.rips.2015.04.002

[B2] Armstrong G. Cain C. Wylie L. Muftiæ L. Bouffard L. (2018). Risk factor profile of youth incarcerated for child to parent violence: a nationally representative sample. *J. Crim. J.* 58 1–9. 10.1016/j.jcrimjus.2018.06.002

[B3] Ato M. López-García J. J. Benavente A. (2013). Un sistema de clasificación de los diseños de investigación en psicología [A classification system for research designs in psychology]. *Anal. Psicol.* 29 1038–1059. 10.6018/analesps.29.3.178511

[B4] Brezina T. (1999). Teenage violence towards parents as an adaptation to family strain. *Youth Soc.* 30 416–444. 10.1177/0044118X99030004002

[B5] Bronfenbrenner U. (1979). *The Ecology of Human Development: Experiments by Nature and Design.* Cambridge, MA: Harvard University Press.

[B6] Brule N. J. Eckstein J. J. (2016). “Am i really a bad parent?”: adolescent-to-parent abuse (AtPA) identity and the stigma management communication (SMC) model. *J. Family Commun.* 16 198–215. 10.1080/15267431.2016.1160908

[B7] Calvete E. Pereira R. (2019a). “Conceptualización de la violencia filioparental, magnitud y teorías explicativas [Conceptualization of child-to-parent violence, magnitude and explicative theories],” in *La Violencia Filio-Parental. Análisis, Evaluación e Intervención*, Vol. 1 eds Calvete E. Pereira R. (Madrid: Alianza), 19–48.

[B8] Calvete E. Pereira R. (2019b). *La violencia filio-parental. Análisis, evaluación e intervención [Child-to-parent violence. Analysis, evaluation and intervention].* Madrid: Alianza.

[B9] Carrasco N. García J. Zaldívar F. (2018). Diferencias asociadas a la violencia filio-parental en función del tipo de familia (“normalizadas” vs “en riesgo”) y parentesco de la víctima. *Revist. Psicol. Clín. Niños Adolesc.* 5 30–35. 10.21134/rpcna.2018.05.3.4

[B10] Castro-Sánchez M. Zurita-Ortega F. Ruiz G. R.-R. Chacón-Cuberos R. (2019). Explanatory model of violent behaviours, self-concept and empathy in schoolchildren. Structural equations analysis. *PLoS One* 14:e0217899. 10.1371/journal.pone.0217899 31419233 PMC6697324

[B11] Condry R. Miles C. (2014). Adolescent to parent violence: framing and mapping a hidden problem. *Criminol. Crim. Justice* 14 257–275. 10.1177/1748895813500155

[B12] Contreras L. Cano C. (2014a). Adolescents who assault their parents: a different family profile of young offenders? *Violence Victims* 29 393–406. 10.1891/0886-6708.VV-D-12-00132 25069145

[B13] Contreras L. Cano C. (2014b). Family profile of young offenders who abuse their parents: A comparison with general offenders and non-offenders. *J. Family Violence* 29 901–910. 10.1007/s10896-014-9637-y 25069145

[B14] Contreras L. Cano C. (2015). Exploring psychological features in adolescents who assault their parents: a different profile of young offenders? *J. Forensic Psychiatry Psychol.* 26 224–241. 10.1080/14789949.2015.1004634

[B15] Contreras L. Cano C. (2016a). Child-to-parent violence: The role of exposure to violence and its relationship to social-cognitive processing. *Eur. J. Psychol. Appl. Legal Context* 8 43–50. 10.1016/j.ejpal.2016.03.003

[B16] Contreras L. Cano C. (2016b). Social competence and child-to-parent violence: analyzing the role of the emotional intelligence, social attitudes, and personal values. *Deviant Behav.* 37 115–125. 10.1080/01639625.2014.983024

[B17] Cortina H. Martín A. M. (2020). The behavioral specificity of child-to-parent violence. *Anal. Psicol.* 36 386–399. 10.6018/analesps.411301

[B18] Cottrell B. (2001). *Parent Abuse: The Abuse of Parents by Their Teenage Children.* Ottawa, ON: The Family Violence Prevention Unit Health, Canada.

[B19] Curtis A. Harries T. Moulds L. Miller P. (2019). Addressing child-to-parent violence: developmental and intervention considerations. *J. Family Stud.* 10.1080/13229400.2019.1682643 [Epub ahead of print].

[B20] Fariña F. Redondo L. Seijo D. Novo M. Arce R. (2017). A meta-analytic review of the MMPI validity scales and indexes to detect defensiveness in custody evaluations. *Int. J. Clin. Health Psychol.* 17 128–138. 10.1016/j.ijchp.2017.02.002 30487888 PMC6220924

[B21] Fleury R. E. Sullivan C. M. Bybee D. I. (2000). When ending the relationship does not end the violence: women’s experiences of violence by former partners. *Violence Against Women* 6 1363–1383. 10.1177/10778010022183695

[B22] Gallego R. Novo M. Fariña F. Arce R. (2019). Child-to-parent violence and parent-to-child violence: a meta-analytic review. *Eur. J. Psychol. Appl. Legal Context* 11 51–59. 10.5093/ejpalc2019a4

[B23] Garaigordobil M. Durá A. (2006). Relaciones del autoconcepto y la autoestima con la sociabilidad, estabilidad emocional y responsabilidad en adolescentes de 14 a 17 años [Relationships of self-concept and self-esteem with sociability, emotional stability and responsibility in adolescent]. *Anál. Mod. Conducta* 32 37–64. 10.33776/amc.v32i141.2132

[B24] García F. Musitu G. (2014). *Autoconcepto Forma 5[Sef-concept Form 5] (4th edition, revised).* Madrid: Tea.

[B25] Hernández A. (2016). *El perfil psicosocial de los agresores y de las víctimas de la violencia filioparental [Psychosocial profile of aggressors and victims of child-to-parent violence].* Doctoral Thesis, Universidad de La Laguna, San Cristóbal de La Laguna.

[B26] Hollenstein T. Lougheed J. P. (2013). Beyond storm and stress: typicality, transactions, timing, and temperament to account for adolescent change. *Am. Psychol.* 68 444–454. 10.1037/a0033586 23915399

[B27] Holt A. (2016). Adolescent-to-parent abuse as a form of “domestic violence” a conceptual review. *Trauma Violence Abuse* 17 490–499. 10.1177/1524838015584372 25971709

[B28] Holt A. Retford S. (2013). Practitioner accounts of responding to parent abuse—A case study in ad hoc delivery, perverse outcomes and a policy silence. *Child Family Soc. Work* 18 365–374. 10.1111/j.1365-2206.2012.00860.x

[B29] Hong J. S. Kral M. J. Espelage D. L. Allen-Meares P. (2012). The social ecology of adolescent-initiated parent abuse: a review of the literature. *Child Psychiatry Hum. Dev.* 43 431–454. 10.1007/s10578-011-0273-y 22160270

[B30] Hoyo-Bilbao J. D. Orue I. Gámez-Guadix M. Calvete E. (2020). Multivariate models of child-to-mother violence and child-to-father violence among adolescents. *Eur. J. Psychol. Appl. Legal Context* 12 11–21. 10.5093/ejpalc2020a2

[B31] Ibabe I. (2015). Family predictors of child-to-parent violence: the role of family discipline. *Anal. Psicol.* 31 615–625. 10.6018/analesps.31.2.174701

[B32] Ibabe I. Arnoso A. Elorriaga E. (2014). Behavioral problems and depressive symptomatology as predictors of child-to-parent violence. *Eur. J. Psychol. Appl. Legal Context* 6 53–61. 10.1016/j.ejpal.2014.06.004

[B33] Ibabe I. Arnoso A. Elorriaga E. (2018). Programas de intervención destacados en violencia filio-parental: Descripción de un programa innovador de intervención precoz. *Papeles Psicól.* 39 208–217. 10.23923/pap.psicol2018.2873

[B34] Ibabe I. Jaureguizar J. Díaz O. (2009). Adolescent violence against parents. Is it a consequence of gender inequality? *Eur. J. Psychol. Appl. Legal Context* 1 3–24.

[B35] IBM Corporation (2019). *IBM SPSS Statistics for Windows, Version 26.0.* Armonk, NY: IBM Corporation.

[B36] Kennedy T. D. Edmonds W. A. Dann K. T. J. Burnett K. F. (2010). The clinical and adaptive features of young offenders with histories of child-parent violence. *J. Family Violence* 25 509–520. 10.1007/s10896-010-9312-x

[B37] Kowalski R. M. Limber S. P. (2013). Psychological, physical, and academic correlates of cyberbullying and traditional bullying. *J. Adolesc. Health* 53:20. 10.1016/j.jadohealth.2012.09.018 23790195

[B38] León C. (2019). Family communication patterns, school and family self-concept, and motivation of revenge among adolescents. *Eur. J. Invest. Health Psychol. Educ.* 9 51–58. 10.30552/ejihpe.v9i1.316

[B39] Loinaz I. Echeburua E. Ortiz-Tallo M. Amor P. J. (2012). Psychometric properties of the Conflict Tactics Scales (CTS-2) in a Spanish sample of partner-violent men. *Psicothema* 24 142–148. 22269377

[B40] Martín A. M. Padrón F. Redondo S. (2019). Early narratives of desistance from crime in different prison regimes. *Eur. J. Psychol. Appl. Legal Context* 11 71–79. 10.5093/ejpalc2019a2

[B41] Memoria de la Fiscalía General del Estado (2019). *[Report of the Attorney General’s Office].* Available online at: https://d3cra5ec8gdi8w.cloudfront.net/uploads/documentos/2019/09/10/_memoria2019_76609dd4.pdf (accessed July 1, 2020).

[B42] Moreno D. Estévez E. Murgui S. Musitu G. (2009). Reputación social y violencia relacional en adolescentes: El rol de la soledad, la autoestima y la satisfacción vital [Social reputation and relational aggression in adolescence: the role of loneliness, self-esteem and life satisfaction]. *Psicothema* 21 537–542. 19861095

[B43] Moulds L. Day A. Mayshak R. Mildred H. Miller P. (2018). Adolescent violence towards parents: prevalence and characteristics using Australian Police Data. *Aust. New Zeal. J. Criminol.* 52 231–249. 10.1177/0004865818781206

[B44] Muñoz-Rivas M. J. Andreu R. J. Graña G. J. O’Leary D. K. González M. P. (2007). Validation of the modified version of the Conflict Tactics Scale (M-CTS) in a Spanish population of youths [Validation of the modified version of the conflict tactics scale (M-CTS) in a Spanish Population of Youths]. *Psicothema* 19:693. 17959128

[B45] Orue I. Calvete E. (2010). Elaboración y validación de un cuestionario para medir la exposición a la violencia en infancia y adolescencia [Elaboration and validation of a questionnaire for measuring exposure to violence in infancy and adolescence]. *Int. J. Psychol. Psychol. Ther.* 10 279–292.

[B46] Pagani L. S. Larocque D. Vitaro F. Tremblay R. E. (2003). Verbal and physical abuse toward mothers: the role of family configuration, environment, and coping strategies. *J. Youth Adolesc.* 32 215–223. 10.1023/A:1022599504726

[B47] Pereira R. (2019). “La intervención en violencia filioparental desde el modelo sistémico [Intervention in child-to-parent violence from the systemic model],” in *La Violencia filio-Parental. Análisis, Evaluación e Intervención*, Vol. 6 eds Calvete E. Pereira R. (Madrid: Alianza), 163–202.

[B48] Pérez T. Pereira R. (2006). Violencia filio-parental: revisión de la bibliografía. *Revist. Mosaico* 36 10–17.

[B49] Real Statistics Resource Pack software 7.2 (2013-2020). *Real Statistics Resource Pack software (Release 7.2). Copyright (2013 – 2020) Charles Zaiontz.* Available online at: https://www.real-statistics.com/ (accessed September 30, 2020).

[B50] Romero A. León C. Musitu D. Villarreal M. E. (2019). Family functioning, self-concept and cybervictimization: An analysis based on gender. *Soc. Sci.* 8:69 10.3390/socsci8020069

[B51] Sears R. R. Maccoby E. E. Levin H. (1957). *Patterns of Child Rearing.* Stanford, CA: Stanford University Press.

[B52] Simmons M. McEwan T. Purcell R. Ogloff J. (2018). Sixty years of child-to-parent abuse research: what we know and where to go. *Aggr. Violent Behav.* 38 31–52. 10.1016/j.avb.2017.11.001

[B53] Simmons M. L. McEwan T. E. Purcell R. (2019a). “But all kids yell at their parents, don’t they?”: social norms about child-to-parent abuse in Australia. *J. Family Issues* 40 1486–1508. 10.1177/0192513x19842587

[B54] Simmons M. L. McEwan T. E. Purcell R. Huynh M. (2019b). The abusive behaviour by children-indices (ABC-I): a measure to discriminate between normative and abusive child behaviour. *J. Family Violence* 34 663–676. 10.1007/s10896-019-00071-1

[B55] Straus M. A. (1979). Measuring intrafamily conflict and violence: the conflict tactics (CT) scales. *J. Marriage Family* 41 75–88. 10.2307/351733

[B56] Straus M. A. Hamby S. L. Finkelhor D. Moore D. W. Runyan D. (1998). Identification of child maltreatment with the parent-child conflict tactics scales: development and psychometric data for a national sample of American parents. *Child Abuse Neglect* 22 249–270. 10.1016/S0145-2134(97)00174-9 9589178

[B57] Vilariño M. Arce R. Fariña F. (2013). Forensic-clinical interview: Reliability and validity for the evaluation of psychological injury. *Eur. J. Psychol. Appl. Legal Context* 5 1–21.

[B58] Williams M. Tuffin K. Niland P. (2016). “It’s like he just goes off, boom!”: mothers and grandmothers make sense of child-to-parent violence. *Child Family Soc. Work* 22 597–606. 10.1111/cfs.12273

